# Seroprevalence and Risk Factors for Human Herpesvirus 8 Infection, Rural Egypt^1^

**DOI:** 10.3201/eid1404.070935

**Published:** 2008-04

**Authors:** Sam M. Mbulaiteye, Ruth M. Pfeiffer, Bryan Dolan, Victor C.W. Tsang, John Noh, Nabiel N.H. Mikhail, Mohamed Abdel-Hamid, Mohamed Hashem, Denise Whitby, G. Thomas Strickland, James J. Goedert

**Affiliations:** * National Cancer Institute, Bethesda, Maryland, USA; † Centers for Disease Control and Prevention, Atlanta, Georgia, USA; ‡ Assiut University, Cairo, Egypt; § National Hepatology and Tropical Medicine Research Institute, Cairo, Egypt; ¶ University of Maryland School of Medicine, Baltimore, Maryland, USA; # National Cancer Institute-Frederick, Frederick, MD, USA

**Keywords:** HHV-8, epidemiology, transmission, Africa, cancer, schistosomiasis, research

## Abstract

To determine whether human herpesvirus 8 (HHV-8) is associated with schistosomal and hepatitis C virus infections in Egypt, we surveyed 965 rural household participants who had been tested for HHV-8 and schistosomal infection (seroprevalence 14.2% and 68.6%, respectively, among those <15 years of age, and 24.2% and 72.8%, respectively, among those ≥15 years of age). Among adults, HHV-8 seropositivity was associated with higher age, lower education, dental treatment, tattoos, >10 lifetime injections, and hepatitis C virus seropositivity. In adjusted analyses, HHV-8 seropositivity was associated with dental treatment among men (odds ratio [OR] 2.4, 95% confidence interval [CI] 1.1–5.2) and hepatitis C virus seropositivity among women (OR 3.3, 95% CI 1.4–7.9). HHV-8 association with antischistosomal antibodies was not significant for men (OR 2.1, 95% CI 0.3–16.4), but marginal for women (OR 1.5, 95% CI 1.0–2.5). Our findings suggest salivary and possible nosocomial HHV-8 transmission in rural Egypt.

## Abstract 2

Seroprevalence and Risk Factors for Human Herpesvirus 8 Infection, Rural Egypt, Salivary and nosocomial transmission are possible.

Human herpesvirus 8 (HHV-8, also called Kaposi sarcoma [KS]–associated herpesvirus) is the infectious cause of KS (*1*) and is prevalent in Africa (*2*). HHV-8 seroepidemiology parallels imperfectly KS epidemiology (*3*). Adult HHV-8 seropositivity is very high in eastern and central Africa (70%–90%), where KS is endemic, and lower in southern and northern Africa (10%–40%), including Egypt, where KS is more rare (*4*). This variation may be due, in part, to socioeconomic or environmental factors (*5*) influencing HHV-8 transmission or pathogenesis. HHV-8 is transmitted through contact with saliva (*6**–**8*), but sexual (*9*) and blood-borne (*10*) transmission also occur.

HHV-8 seroepidemiology in Egypt is incompletely described (*3*,*11*,*12*). Egypt offers the opportunity to investigate how HHV-8 correlates in the general population with well-characterized hepatitis C virus (HCV) (*13*) and schistosomal infections (*14*). Hundreds of thousands of Egyptians were exposed to multiple intravenous injections during treatment campaigns to control schistosomiasis from the 1950s until 1982, which resulted in an epidemic of HCV (*15*). Schistosomal infection has been reported to suppress the immune response to HCV, which could lead to more persistent infections in those who are co-infected (*16*–*20*). Whether a similar relationship exists with HHV-8 is not known. The rarity of KS in Egypt, despite its occurrence in organ transplant recipients (*21*), suggests that schistosomal-induced immunosuppression may not increase KS risk substantially. We sought to test the hypothesis that schistosomal seropositivity is associated with HHV-8 seropositivity, which would support the concept that schistosomal-induced immunosuppression modulates susceptibility to HHV-8 infection.

## Methods

### Patient Selection and Serologic Testing for HHV-8 and Schistosomal Infections

We randomly selected residual frozen serum samples from 965 of ≈6,000 persons who had participated in the HCV and schistosomiasis epidemiologic, population–based, household survey in Assiut Governorate, in rural southern Egypt in 1992 (*13*). Adults and parents of children <15 years of age who had participated in the original survey gave informed consent and answered interviewer-administered questions about demographics, socioeconomic status, medical treatment, and parenteral exposures, including injections, transfusions, operations, dental treatment, and tattoos. Each participant gave a venous blood sample for serologic testing (*13*). The proportion of children included in our study was slightly lower than in the original survey because serum samples from some children had been exhausted by tests for HCV and other hepatitis viruses. However, the included children were otherwise representative of the original survey population.

Anti–HHV-8 antibodies were measured by using an enzyme immunoassay to K8.1 glycoprotein (a lytic-phase antigen) as previously reported (*22*,*23*). Antischistosomal antibodies were measured by using enzyme-linked immunoelectrotransfer blots (EITB) to detect species-specific antibodies against microsomal glycoprotein antigens from *Schistosoma hematobium* and *S. mansoni* (98% sensitivity and 99% specificity) (*24*). *S. hematobium* is the predominant species causing infection in Assiut Governorate; concurrent or single infection with *S. mansoni* among local inhabitants is rare (*14*).

### Statistical Methods

Because no standard for testing for HHV-8 infection exists, we previously based cutoff values for defining seropositivity on visual inspection of the distribution of the K8.1 assay optical density (OD) values (*10*), assuming that there are seronegative and seropositive subpopulations. However, this approach is subjective. To more objectively define seropositivity, we applied mixture models to the OD data (*25*). Briefly, the mixture model was based on the assumption that the OD value for each participant arises from either a seronegative or a seropositive subpopulation. The formulas and details on parameters for the statistical model are described elsewhere ([*25*]; online Appendix, available from www.cdc.gov/EID/content/14/4/586-app.htm). We assumed that when the calculated posterior probability of seropositivity was >0.5, then the person was seropositive. In sensitivity analyses (online Appendix, available from www.cdc.gov/EID/content/14/4/586-app.htm), we used alternative parameters of the model and also excluded persons with an intermediate posterior probability (range: 0.4–0.6).

After defining seropositivity, we used logistic regression models (PROC GENMOD, SAS 8.0; SAS, Cary, NC, USA) to calculate odds ratios (ORs) for HHV-8 associations with demographic, behavioral, and clinical risk factors. We used generalized estimation equations that accounted for correlations between persons living in the same household to calculate 95% confidence intervals (CIs) (*26*). Because HHV-8 seropositivity is age dependent and modes of transmission may differ between children and adults, we performed univariate and multivariable analyses separately for children (<15 years of age) and adults (>15 years of age). Because we postulated a priori that antischistosomal antibodies were associated with HHV-8, we included schistosomal status in all multivariable models. To adjust for potential confounding, we included in our multivariable models those variables that were associated with HHV-8 seropositivity at p<0.1 in univariate analyses. Age was fitted with a trend whenever this resulted in a statistically significantly improved model fit; otherwise, it was fitted as a categoric variable with dummy values. Two-tailed p values (p<0.05) were considered statistically significant, while p values between 0.1 and 0.05 were suggestive of a trend.

## Results

None of the original household survey participants had a history of KS (*13*). HHV-8 seroprevalence was lower among children compared with adults (14.3% vs. 24.2%, p<0.001). Among children, in unadjusted analyses, HHV-8 seroprevalence was higher in girls than boys (20% vs. 9%; OR 2.4, 95% CI 1.1–5.3). HHV-8 seroprevalence was not significantly elevated in children with a history of >10 lifetime injections compared with those with <10 lifetime injections (18% vs. 11%, OR 1.8, 95% CI 0.8–3.8) nor among those with schistosomal antibodies (16% vs. 10%; OR 1.7, 95% CI 0.7–4.3). Age, education, dental treatment, tattoos, and HCV antibodies were unrelated to HHV-8 seropositivity among children. 

Among adult men and women combined, unadjusted analyses showed that HHV-8 seropositivity was higher among older participants (>45 years of age) compared with younger participants (15–24 years of age; OR 4.1, 95% CI 2.6–6.6); among those currently married (OR 1.9, 95% CI 1.2–3.0) or divorced, separated, or widowed (OR 3.3, 95% CI 1.7–6.4) versus never married; and among those with a history of dental treatment (OR 2.1, 95% CI 1.5–2.9), >10 lifetime injections (OR 1.5, 95% CI 1.0–2.3), tattoos (OR 1.7, 95% CI 1.1–2.7), or HCV seropositivity (OR 1.8, 95% CI 1.0–3.3) compared with participants without these characteristics. Conversely, HHV-8 seropositivity was lower among adults who reported primary (OR 0.6, 95% CI 0.4–1.0) or higher level of formal education (OR 0.3, 95% CI 0.2–0.6) compared with participants without formal education.

Antischistosomal antibodies, which indicate past as well current infection, were detected among 72.8% of participants. Current infection, as indicated by ova in stool or urine samples, was noted for 4% of participants, all of whom had *S. hematobium*. *S. mansoni* was only recently introduced in Assiut Governorate and remains rare and focal in distribution (*14*). Almost all participants who had *S. mansoni* antibodies were also positive for *S. hematobium*. Patterns of schistosomal seropositivity differed between women and men. Among women, antischistosomal antibody prevalence was inversely related with age (68.8% in those 15–24 years of age vs. 34.1% in those >45 years of age; p<0.001); was lower in those who were widowed, divorced, or separated compared with those who were married or who had never married (38.8% vs. 63.4%; p = 0.003); but was unrelated to education (p = 0.36) or HCV seropositivity (p = 0 .12). Among men, antischistosomal antibody prevalence was unrelated to age (p = 0.61), marital status (p = 0.73), education (p = 0.64), or HCV seropositivity (p = 0.12).

In unadjusted sex-specific analyses (Tables 1 and 2), HHV-8 seropositivity was not associated with antischistosomal antibodies in women (OR 1.0, 95% CI 0.6–1.5); it was not significantly associated in men (OR 2.3, 95% CI 0.3–19.0), because only 9 men had no antischistosomal antibodies. The HHV-8 associations with age, formal education, marital status, and history of dental treatment among men and women combined were largely recapitulated in the sex-specific analyses, except for associations with having tattoos, >10 lifetime injections, and HCV seropositivity, which were evident in women but not men (Tables 1 and 2). HHV-8 was significantly associated with cigarette smoking; cigarette smoking was only recorded for men.

**Table 1:** Prevalence and crude OR of association of HHV-8 seropositivity with demographic, behavioral, and clinical characteristics among male patients, Egypt*

Characteristic	n/N	%	OR	95% CI	p value†
Total	52/235	22.1	–	–	–
Age group, y					<0.001
15–24	8/96	8.3	Ref	–	–
25–34	16/54	29.6	4.6	2.0–11.0	–
35–44	10/30	33.3	5.5	1.9–15.7	–
>45	18/55	32.7	5.3	2.2–13.2	–
Education					0.001
None	19/50	38.0	Ref	–	–
Primary	22/100	22.0	0.5	0.2–1.0	–
Secondary/postsecondary	11/85	12.9	0.2	0.1–0.6	–
Job					0.001
Student	2/38	5.3	Ref	–	–
Not working	6/31	19.3	4.3	0.8–23.3	–
Farmer	25/87	28.7	7.3	1.6–32.4	–
Trade, service, production	13/63	20.6	4.7	1.1–20.0	–
Technician/secretary	6/16	37.5	10.8	1.9–61.3	–
Marital status					0.004
Not married	11/91	12.1	Ref	–	–
Married	39/136	28.7	3.1	1.4–6.5	–
Separated/divorced/widowed	2/8	25.0	7.6	1.0–60.8	–
Dental treatments					<0.001
No	16/128	12.5	Ref	–	–
Yes	36/107	33.6	3.5	1.8–7.1	–
Tattoos					0.53
No	50/220	22.7	Ref	–	–
Present	2/15	13.3	0.5	0.1–2.5	–
HCV serostatus					0.72
Negative	48/214	22.4	Ref	–	–
Positive	4/21	19.0	0.8	0.3–2.5	–
Injections (lifetime)					0.74
<10	20/72	20.8	Ref	–	–
>10	37/163	22.7	1.1	0.6–2.1	–
Smoking					0.02
Never	23/138	16.7	Ref	–	–
Ever	29/97	29.9	2.1	1.1–4.0	–
Goza‡					0.77
Never	42/193	21.8	Ref	–	–
Ever	10/42	23.8	1.1	0.5–2.4	–
Schistosomiasis					0.43
Negative	1/9	11.1	Ref	–	–
Positive	51/226	31.0	2.3	0.3–19.0	–

*OR, odds ratio; HHV-8, human herpesvirus 8; CI, confidence interval; Ref, referrent; HCV, hepatitis C virus. †p value for age group and education is test for trend; otherwise, p value is test for heterogeneity ‡Goza is a method of tobacco smoking in which tobacco smoke passes through a water pipe.

**Table 2:** Prevalence and crude OR of association of HHV-8 seropositivity with demographic, behavioral, and clinical characteristics among female patients, Egypt*

Characteristic	n/N	%	OR	95% CI	p value†
Total	125/495	25.3	–	–	–
Age group, y					<0.001
15–24	23/148	15.5	Ref	–	–
25–34	21/106	19.8	1.4	0.7–2.7	–
35–44	31/115	27.0	2.0	1.1–3.6	–
>45	50/126	39.7	3.6	2.1–6.3	–
Education					<0.001
None	88/307	28.7	Ref	–	–
Primary	34/154	32.7	0.7	0.4–1.1	–
Secondary/postsecondary	4/38	22.5	0.3	0.1–0.8	–
Job					0.11
Other‡	2/21	9.5	Ref	–	–
Housewife	121/470	25.7	3.3	0.8–14.3	–
Dental treatments					0.02
No	49/239	20.5	Ref	–	–
Yes	77/260	29.6	1.6	1.1–2.5	–
Tattoos					0.006
No	93/410	22.7	Ref	–	–
Present	33/89	37.1	2.0	1.2–3.3	–
HCV serostatus					0.006
Negative	112/470	23.8	Ref	–	–
Positive	14/29	48.3	3.0	1.4–6.5	–
Injections (lifetime)					0.03
<10	23/128	18.0	Ref	–	–
>10	103/371	27.8	1.7	1.1–2.9	–
Schistosomiasis					0.94
Negative	48/189	25.4	Ref	–	–
Positive	76/303	25.1	1.0	0.6–1.5	–

*OR, odds ratio; HHV-8, human herpesvirus 8; CI, confidence interval; Ref, referrent; HCV, hepatitis C virus. †p value for age group and education is test for trend; otherwise, p value is test for heterogeneity. ‡Job category for women: “Other” includes women who are students, are not working, or have technical or scientific jobs.

In a multivariable analysis, HHV-8 seropositivity was higher among girls than boys (OR 2.6, 95% CI 1.2–5.8) and marginally associated with antischistosomal antibodies (OR 2.2, 95% CI 0.8–5.6). Among adult men, HHV-8 seropositivity was independently associated with older age and history of dental treatment but not with schistosomal antibodies (OR 2.3, 95% CI 0.3–16.1; Table 3). Among women, HHV-8 seropositivity was associated with older age, HCV seropositivity, and marginally with antischistosomal antibodies (OR 1.5, 95% CI 1.0–2.5; p = 0.07).

**Table 3:** Adjusted OR of association of HHV-8 seropositivity with demographic and clinical variables among adults, Egypt*

Characteristic	Men				Women		
	OR	95% CI	p value		OR	95% CI	p value
Age group, y†			0.002				
15–24	Ref	–	–		Ref	–	–
25–34	1.6	1.2–2.2	–		0.8	0.4–1.6	0.53
35–44	2.6	1.4–4.9	–		1.5	0.8–2.9	0.15
>45	4.3	1.7–11.0	–		3.1	1.5–6.4	<0.001
Dental treatments‡			0.04				
No	Ref	–	–		–	–	–
Yes	2.3	1.1–4.9	–		–	–	–
HCV serostatus‡			–				0.007
Negative	–	–	–		Ref	–	–
Positive	–	–	–		3.3	1.4–7.9	–
Schistosomiasis§			0.47				0.07
Negative	Ref	–	–		Ref	–	–
Positive	2.3	0.3–16.1	–		1.5	1.0–2.5	–

*OR, odds ratio; HHV-8, human herpesvirus 8; CI, confidence interval; Ref, referrent; HCV, hepatitis C virus. †p value is for age group fitted with trend among men; p values for heterogeneity for categories given for women (see Statistical Methods). ‡Missing values in sex-specific analyses mean the variable was not significant and was excluded from final multivariable model. §Schistosomiasis seropositivity was included in models even when not significant because we hypothesized a priori that it was associated with HHV-8 seropositivity (see online Appendix, available from www.cdc.gov/EID/content/14/4/586-app.htm).

## Discussion

We report HHV-8 seroepidemiology in a rural population in Egypt in which correlates of schistosomal and HCV were previously characterized (*13**,**14*). As in other populations, HHV-8 seropositivity in Egypt rose with increasing age (*3**,**5*). In subgroups, we found associations of HHV-8 seropositivity with history of dental therapy, lifetime injections, tattoos, and HCV seropositivity.

Previous studies of HHV-8 in Egypt (*11*,*12*) reported a seroprevalence of ≈40%, which is ≈2× the prevalence we observed. Those studies were hospital based, were conducted in urban areas, and detected anti-HHV-8 antibodies with lytic immunofluorescence assays; all of these factors may have contributed to higher prevalence estimates. Despite these differences, the patterns in our associations support their validity. First, we detected HHV-8 antibodies in children, consistent with earlier reports and nonsexual HHV-8 spread in Egypt (*11*). Second, HHV-8 seroprevalence increased with age, in accord with the general pattern of HHV-8 observed in other populations (*5*). Among men, HHV-8 seropositivity was significantly associated with dental treatment, which may be a marker for transmission through saliva- or blood-contaminated dental instruments. Our HHV-8 associations with a history of >10 lifetime injections, tattoos, and HCV seropositivity also point to possible blood-borne transmission and agree with other studies (*10*,*27*,*28*). Sexual transmission might be suggested by our HHV-8 association with marital status, but this association did not persist after adjustment for age. Rather than ongoing transmission among adults, higher HHV-8 seropositivity in older persons may be due to a birth-cohort effect, i.e., reflecting periods of elevated HHV-8 transmission risk in the past. In this regard, the widespread use of intravenous injections for schistosomiasis treatment and control programs from 1950 to 1982 would be consistent with a birth-cohort effect for high HHV-8 seroprevalence among our older participants. However, most populations, including those with no similar historical programs, have higher HHV-8 seroprevalence among older persons, which suggests that ongoing HHV-8 transmission among adults is a more likely explanation.

We found a 2-fold higher HHV-8 seroprevalence in persons who also had schistosomal antibodies. In multivariable analyses, the CI for this association was 1.0–2.5 among women, but it was wide among children (0.8–5.6) and especially among men (0.3–16.1). Chance association and serologic cross-reactivity between HHV-8 and schistosomal antibodies are possible explanations, but schistosomal antibodies may be a valid marker for exposure to injections in the historical anti-schistosomal program; these findings are consistent with parenteral transmission of HHV-8 as discussed above. Another possibility is that anti-schistosomal antibodies may be a marker for contact with surface water sources or walking barefoot, which are also risk factors for HHV-8 seropositivity and KS (*5*,*7*,*29*). No biologic explanation has been advanced for these environmental correlations with HHV-8 and KS. Our study suggests that perhaps contact with surface water or walking barefoot is a marker of exposure to and potential infection with *Schistosoma* or other water-related parasites. Infection with such parasites could influence the natural history of HHV-8 by shifting the immune response from a T helper 1 (Th1)–type response, which is central to controlling viral infections, to a Th2-dominant response (*30*), which is less effective against viral infections. If this model is correct, schistosomal infection could increase susceptibility to HHV-8 infection at relatively low exposure to the virus. In parallel, Th2-dominant hosts may fail to effectively control HHV-8 infection and thus shed infectious virions in saliva more frequently and at higher levels, resulting in higher HHV-8 transmission. If our findings are confirmed, they could drive investigations of environmental characteristics, including exposures to volcanic soil or plants (*31*), to explain variation in HHV-8 infection and possibly KS.

Our study has several limitations. First, current HHV-8 serologic assays have imperfect specificity and sensitivity (*32*), which could have contributed to the lower HHV-8 seroprevalence observed. Except for the possible cross-reactivity mentioned above, serologic misclassification is likely to be random, which would attenuate associations toward the null. Second, our HCV and schistosomal antibody assays cannot distinguish current from resolved infections, diminishing the strength of observed associations as well. Third, with our cross-sectional design, we cannot determine the temporality of associations. This limitation may be particularly relevant to our findings of HHV-8 with antischistosomal antibodies. The antischistosomal programs surely reduced the prevalence and load of schistosoma eggs, but they may also have contributed to HHV-8 transmission through injections. Finally, we studied only ≈15% of the participants in the original survey, which limited our statistical power to estimate some associations. 

The strengths of our study include our state-of-the-art serologic methods, our model-based approach to estimating infection risk, and our well-characterized general population with detailed socioeconomic and clinical data.HHV-8 seropositivity was associated with older age, dental therapy, lifetime injections, and HCV and schistosomiasis seropositivity. These findings suggest salivary and possible nosocomial HHV-8 transmission in rural Egypt and a potential biologic explanation for geographic variation of HHV-8 seropositivity and KS.

## Supplementary Material

Appendix

## References

[1] Chang Y, Cesarman E, Pessin MS, Lee F, Culpepper J, Knowles DM, Identification of herpesvirus-like DNA sequences in AIDS-associated Kaposi’s sarcoma. Science. 1994;266:1865–9. 10.1126/science.7997879 7997879

[2] Boshoff C, Weiss RA. Epidemiology and pathogenesis of Kaposi’s sarcoma–associated herpesvirus. Philos Trans R Soc Lond B Biol Sci. 2001;356:517–34. 11313009 10.1098/rstb.2000.0778 PMC1088442

[3] Dedicoat M, Newton R. Review of the distribution of Kaposi’s sarcoma-associated herpesvirus (KSHV) in Africa in relation to the incidence of Kaposi’s sarcoma. Br J Cancer. 2003;88:1–3. 10.1038/sj.bjc.6600745 12556950 PMC2376771

[4] Dukers NH, Rezza G. Human herpesvirus 8 epidemiology: what we do and do not know. AIDS. 2003;17:1717–30. 10.1097/00002030-200308150-00001 12891058

[5] Mbulaiteye SM, Biggar RJ, Pfeiffer RM, Bakaki PM, Gamache C, Owor AM, Water, socioeconomic factors, and human herpesvirus 8 infection in Ugandan children and their mothers. J Acquir Immune Defic Syndr. 2005;38:474–9. 10.1097/01.qai.0000132495.89162.c0 15764964

[6] Plancoulaine S, Abel L, van Beveren M, Tregouet DA, Joubert M, Tortevoye P, Human herpesvirus 8 transmission from mother to child and between siblings in an endemic population. Lancet. 2000;356:1062–5. 10.1016/S0140-6736(00)02729-X 11009141

[7] Mbulaiteye SM, Pfeiffer RM, Whitby D, Brubaker GR, Shao J, Biggar RJ. Human herpesvirus 8 infection within families in rural Tanzania. J Infect Dis. 2003;187:1780–5. 10.1086/374973 12751036

[8] Bourboulia D, Whitby D, Boshoff C, Newton R, Beral V, Carrara H, Serologic evidence for mother-to-child transmission of Kaposi sarcoma–associated herpesvirus infection. JAMA. 1998;280:31-a–2. 10.1001/jama.280.1.31-a 9660357

[9] Eltom MA, Mbulaiteye SM, Dada AJ, Whitby D, Biggar RJ. Transmission of human herpesvirus 8 by sexual activity among adults in Lagos, Nigeria. AIDS. 2002;16:2473–8. 10.1097/00002030-200212060-00014 12461423

[10] Mbulaiteye SM, Biggar RJ, Bakaki PM, Pfeiffer RM, Whitby D, Owor AM, Human herpesvirus 8 infection and transfusion history in children with sickle-cell disease in Uganda. J Natl Cancer Inst. 2003;95:1330–5. 12953087 10.1093/jnci/djg039

[11] Andreoni M, Sarmati L, Nicastri E, El Sawaf G, El Zalabani M, Uccella I, Primary human herpesvirus 8 infection in immunocompetent children. JAMA. 2002;287:1295–300. 10.1001/jama.287.10.1295 11886321

[12] Serraino D, Toma L, Andreoni M, Butto S, Tchangmena O, Sarmati L, A seroprevalence study of human herpesvirus type 8 (HHV8) in eastern and central Africa and in the Mediterranean area. Eur J Epidemiol. 2001;17:871–6. 10.1023/A:1015678312153 12081107

[13] Nafeh MA, Medhat A, Shehata M, Mikhail NN, Swifee Y, Abdel-Hamid M, Hepatitis C in a community in Upper Egypt: I. Cross-sectional survey. Am J Trop Med Hyg. 2000;63:236–41. 11421370

[14] Hammam HM, Allam FA, Moftah FM, Abdel-Aty MA, Hany AH, Abd-El-Motagaly KF, The epidemiology of schistosomiasis in Egypt: Assiut Governorate. Am J Trop Med Hyg. 2000;62:73–9. 10813503 10.4269/ajtmh.2000.62.2_suppl.10813503

[15] Frank C, Mohamed MK, Strickland GT, Lavanchy D, Arthur RR, Magder LS, The role of parenteral antischistosomal therapy in the spread of hepatitis C virus in Egypt. Lancet. 2000;355:887–91. 10.1016/S0140-6736(99)06527-7 10752705

[16] Kamal S, Madwar M, Bianchi L, Tawil AE, Fawzy R, Peters T, Clinical, virological and histopathological features: long-term follow-up in patients with chronic hepatitis C co-infected with *S. mansoni.* Liver. 2000;20:281–9. 10.1034/j.1600-0676.2000.020004281.x 10959806

[17] Kamal SM, Rasenack JW, Bianchi L, Al Tawil A, El Sayed Khalifa K, Peter T, Acute hepatitis C without and with schistosomiasis: correlation with hepatitis C-specific CD4(+) T-cell and cytokine response. Gastroenterology. 2001;121:646–56. 10.1053/gast.2001.27024 11522749

[18] Kamal SM, Bianchi L, Al Tawil A, Koziel M, El Sayed Khalifa K, Peter T, Specific cellular immune response and cytokine patterns in patients coinfected with hepatitis C virus and *Schistosoma mansoni.* J Infect Dis. 2001;184:972–82. 10.1086/323352 11574911

[19] Kamal SM, Graham CS, He Q, Bianchi L, Tawil AA, Rasenack JW, Kinetics of intrahepatic hepatitis C virus (HCV)-specific CD4+ T cell responses in HCV and *Schistosoma mansoni* coinfection: relation to progression of liver fibrosis. J Infect Dis. 2004;189:1140–50. 10.1086/382278 15031780

[20] Farid A, Al-Sherbiny M, Osman A, Mohamed N, Saad A, Shata MT, Schistosoma infection inhibits cellular immune responses to core HCV peptides. Parasite Immunol. 2005;27:189–96. 10.1111/j.1365-3024.2005.00762.x 15987342 PMC3906676

[21] El-Agroudy AE, El-Baz MA, Ismail AM, Ali-El-Dein B, El-Dein AB, Ghoneim MA. Clinical features and course of Kaposi’s sarcoma in Egyptian kidney transplant recipients. Am J Transplant. 2003;3:1595–9. 10.1046/j.1600-6135.2003.00276.x 14629292

[22] Engels EA, Sinclair MD, Biggar RJ, Whitby D, Ebbesen P, Goedert JJ, Latent class analysis of human herpesvirus 8 assay performance and infection prevalence in sub-Saharan Africa and Malta. Int J Cancer. 2000;88:1003–8. 10.1002/1097-0215(20001215)88:6<1003::AID-IJC26>3.0.CO;2-9 11093828

[23] Engels EA, Whitby D, Goebel PB, Stossel A, Waters D, Pintus A, Identifying human herpesvirus 8 infection: performance characteristics of serologic assays. J Acquir Immune Defic Syndr. 2000;23:346–54. 10836758 10.1097/00126334-200004010-00011

[24] Al-Sherbiny MM, Osman AM, Hancock K, Deelder AM, Tsang VC. Application of immunodiagnostic assays: detection of antibodies and circulating antigens in human schistosomiasis and correlation with clinical findings. Am J Trop Med Hyg. 1999;60:960–6. 10403328 10.4269/ajtmh.1999.60.960

[25] Pfeiffer RM, Carroll RJ, Wheeler W, Whitby D, Mbulaiteye S. Combining assays for estimating prevalence of human herpesvirus 8 infection using multivariate mixture models. Biostatistics. 2007;9:137–51. 10.1093/biostatistics/kxm018 17566074 PMC2710882

[26] Zeger SL, Liang KY. Longitudinal data analysis for discrete and continuous outcomes. Biometrics. 1986;42:121–30. 10.2307/2531248 3719049

[27] Cannon MJ, Dollard SC, Smith DK, Klein RS, Schuman P, Rich JD, Blood-borne and sexual transmission of human herpesvirus 8 in women with or at risk for human immunodeficiency virus infection. N Engl J Med. 2001;344:637–43. 10.1056/NEJM200103013440904 11228278

[28] Goedert JJ, Charurat M, Blattner WA, Hershow RC, Pitt J, Diaz C, Risk factors for Kaposi’s sarcoma–associated herpesvirus infection among HIV-1–infected pregnant women in the USA. AIDS. 2003;17:425–33. 10.1097/00002030-200302140-00017 12556697

[29] Mbulaiteye SM, Pfeiffer RM, Engels EA, Marshall V, Bakaki PM, Owor AM, Detection of Kaposi sarcoma–associated herpesvirus DNA in saliva and buffy-coat samples from children with sickle cell disease in Uganda. J Infect Dis. 2004;190:1382–6. 10.1086/424489 15378429

[30] Maizels RM, Yazdanbakhsh M. Immune regulation by helminth parasites: cellular and molecular mechanisms. Nat Rev Immunol. 2003;3:733–44. 10.1038/nri1183 12949497

[31] Whitby D, Marshall VA, Bagni RK, Miley WJ, McCloud TG, Hines-Boykin R, Reactivation of Kaposi’s sarcoma–associated herpesvirus by natural products from Kaposi’s sarcoma endemic regions. Int J Cancer. 2007;120:321–8. 10.1002/ijc.22205 17066452 PMC2857915

[32] Rabkin CS, Schulz TF, Whitby D, Lennette ET, Magpantay LI, Chatlynne L, Interassay correlation of human herpesvirus 8 serologic tests. HHV-8 Interlaboratory Collaborative Group. J Infect Dis. 1998;178:304–9. 9697708 10.1086/515649

