# Supplementary material for: Seroprevalence and Risk Factors for Human Herpesvirus 8 Infection, Rural Egypt
Source: Emerg Infect Dis. 2008 Apr;14(4):586–91. doi: 10.3201/eid1404.070935 (PMC2570936; doi:10.3201/eid1404.070935)
Supplement: Appendix [file 07-0935_app.pdf]

Appendix

Statistical Methods

Because there is no standard for testing for human herpesvirus 8 (HHV-8) infection, we defined *infection status* by using a statistical mixture, or latent class, model for the optical density (OD) readings of the K8.1 assay (*1*). Briefly, the model considered the OD assay readings for each participant to arise from either an uninfected (I = 0) or an infected (I = 1) population. The probability density function of $y = (x^{\lambda}-1)/\lambda$, where x denoted the assay readings of the K8.1 assay, is $g(y) = pf(y;\alpha_0) + (1-p) f(y;\alpha_1)$. The probability density function $f(y;\alpha_1)$ reflected the infected population, whereas $f(y;\alpha_0)$ was the probability density function corresponding to uninfected. P is the proportion of uninfected persons in the population. Details on parameters of the component densities and the estimation of the parameters are described elsewhere (*1*). Patients were classified as infected, I = 1, if the posterior probability of infection, given x, i.e., $pr(I = 1| x) = pf(y;\alpha_0) / \{ pf(y;\alpha_0) + (1-p) f(y; \alpha_1)\}$ was ≥ 0.5 and as uninfected otherwise. This classification rule minimized the overall misclassification probability based on the mixture model when discriminating infected from uninfected subjects.

The association of HHV-8 seropositivity with demographic, behavioral, and clinical risk factors was determined by fitting logistic regression models to computed odds ratios (OR) for infection status (PROC GENMOD, SAS 8.0, SAS, Cary, NC, USA). Because HHV-8 seropositivity is age dependent, we performed analyses separately for children and adults to minimize confounding from age. In addition, because we found significant differences by sex and age in the seroepidemiology of schistosomiasis (all p valus <0.05), which we had postulated a priori would be associated with HHV-8, we performed overall and sex-specific analyses for the HHV-8 associations among adults. Because many exposures are age dependent, we constructed multivariable models including variables that were significant at $p \leq 0.1$ in univariate analyses separately for children, men, and women to determine the independent contribution of variables to HHV-8 seropositivity. Age was fitted with a trend whenever this resulted in a statistically significant ($p<0.05$) improved model fit; otherwise, it was fitted as a categoric variable with dummy values for the categories. Schistosomal seropositivity was included in all models because we postulated a priori that it was associated with HHV-8 seropositivity. We used generalized

estimation equations to calculate 95% confidence intervals to account for correlations between participants living in the same household (*2*). We assumed an equally correlated working correlation matrix when computing the variances of the OR estimates, but other working correlations yielded similar results. We assumed that a 2-tailed p value <0.05 was statistically significant and that p values between 0.1 and 0.05 represented a trend.

Sensitivity Analyses

Appendix Tables 1–3 show our sensitivity analyses to address the concern that different model parameterizations may give very different estimates of posterior probabilities of infection. Appendix Table 1 shows HHV-8 seropositivity classification based on the mixture models with the Box Cox transformation and a) normal component densities, b) polynomial degree 1 and 2, c) polynomial degree 2, which was used in the paper. The table shows that no large variations in HHV-8 prevalence are observed, and that the classification of infection based on the posterior probabilities of the respective models leads to different classification of at most 4/734 individuals.

Appendix Table 2 addresses the concern that the degree of uncertainty in the models may lead to widely varying estimates of HHV-8 prevalence and thus, widely varying odds ratios in multivariable association models. The adjusted logistic regression associations before and after exclusion of individuals whose posterior probability was between 0.4-0.6 (38/734 subjects) and using HHV-8 status based on classification from 2 other parameterizations are presented in Appendix Tables 2 and 3. These tables show that the results presented in the manuscript do not appear to be overly sensitive to inclusion of “indeterminate range” individuals (Appendix Table 2) nor to the specific parameterization of the mixture model (Appendix Table 3). The different models gave very similar associations. The stability in the results obtained suggests that the associations that we present are likely valid.

Appendix Table 1. Cross-tabulation of classification of patients by different models to estimate human herpesvirus 8 seropositivity*

Model	Negative	Positive	Total
Model II		Model I	
Negative	556	4	560
Positive	0	174	174
Total	556	178	743
Model III		Model II	
Negative	559	0	559
Positive	1	174	175

Total	560	174	734
Model I		Model III	
Negative	556	0	556
Positive	3	175	178
Total	559	175	734

*I: Classification based on posterior probability from model λ = 1, K = 2 (used in this study); II: Classification based on posterior probability from model λ = 1, K = 1; III: Classification based on posterior probability from model λ = 1, K = 0 (Box Cox transformation included, normal component densities).

Appendix Table 2. Adjusted human herpesvirus 8 associations with and without indeterminate participants

	Model I*			Model I†		
Characteristic	OR	95% CI	p value	OR	95% CI	p value
Men						
Age group, y			<0.002			0.002
15–24	Ref			Ref		
25–34	1.6	1.2–2.2		1.6	1.2–2.2	
35–44	2.6	1.4–4.9		2.6	1.4–5.1	
45+	4.3	1.7–11.0		4.2	1.6–11.4	
Dental treatments			<0.04			0.05
No	Ref			Ref		
Yes	2.3	1.1–4.9		2.3	1.0–5.1	
HCV serology			–			
Negative	–			Ref		
Positive	–	–				
Schistosomiasis			0.47			0.43
Negative	Ref			Ref		
Positive	2.3	0.3–16.1		2.1	0.3–15.5	
Women						
Age group, y						
15–24	Ref			Ref		
25–34	0.8	0.4–1.6	0.53	0.9	0.5–1.9	0.9
35–44	1.5	0.8–2.9	0.15	1.7	0.9–3.2	0.1
45+	3.1	1.5–6.4	<0.001	3.7	2.0–6.9	<0.001
Dental treatments			–			
No	–				–	
Yes	–	–			–	
HCV serology			0.007			0.008
Negative	Ref			Ref		
Positive	3.3	1.4–7.9		3.3	1.4–8.0	
Schistosomiasis			0.07			0.09
Negative	Ref			Ref		
Positive	1.5	1.0–2.5		1.5	0.9–2.5	

*Classification based on posterior probability from model λ = 1, K = 2 (used in the paper). OR, odds ratio; CI, confidence interval; Ref, referrent; HCV, hepatitis C virus.
†Results based on statistical model excluding subjects in the model indeterminate range (posterior probability 0.4–0.6).

Appendix Table 3. Adjusted human herpesvirus 8 associations by using alternative models*

	Model II			Model III		
Characteristic	OR	95% CI	p value	OR	95% CI	p value
Men						
Age group, y			<0.002			0.002
15–24	Ref.			Ref.		
25–34	1.6	1.2–2.3		1.6	1.2–2.2	
35–44	2.7	1.4–5.1		2.6	1.4–4.9	
45+	4.5	1.7–11.7		4.3	1.7–11	
Dental treatments			<0.03			0.04
No	Ref			Ref		
Yes	2.4	1.1–5.3		2.3	1.1–4.9	
HCV serology			–			

Negative	–			Ref		
Positive	–	–				
Schistosomiasis			0.43			0.41
Negative	Ref			Ref		
Positive	2.2	0.3–15.7		2.3	0.3–16.1	
Women						
Age group, y						
15–24	Ref.			Ref.		
25–34	0.8	0.4–1.5	0.43	0.8	0.4–1.5	0.43
35–44	1.5	0.8–2.7	0.23	1.5	0.8–2.7	0.23
45+	3.6	2.0–6.5	<0.001	3.6	2.0–6.5	<0.001
Dental treatments			–			
No	–				–	
Yes	–	–			–	
HCV serology			0.02			0.02
Negative	Ref.			Ref		
Positive	2.9	1.2–6.9		2.9	1.2–6.9	
Schistosomiasis			0.07			0.07
Negative	Ref			Ref		
Positive	1.5	1.0–2.5		1.5	0.1–2.5	

*II, classification based on posterior probability from model $\lambda = 1$, K = 1; III, classification based on posterior probability from model $\lambda = 1$, K = 0 (Box Cox transformation included, normal component densities). R, odds ratio; CI, confidence interval; Ref, referrent; HCV, hepatitis C virus.
